# Possible Mechanisms for Adverse Cardiac Events Caused by Exercise-Induced Hypertension in Long-Distance Middle-Aged Runners: A Review

**DOI:** 10.3390/jcm13082184

**Published:** 2024-04-10

**Authors:** Young-Joo Kim, Kyoung-Min Park

**Affiliations:** 1 Department of Exercise Rehabilitation Welfare, Sungshin Women’s University, 34 da-gil, Bomun-ro, Seongbuk-gu, Seoul 02844, Republic of Korea; 2 Division of Cardiology, Department of Internal Medicine, Heart Vascular and Stroke Institute, Samsung Medical Center, Sungkyunkwan University School of Medicine, 81 Irwon-ro, Gangnam-gu, Seoul 06351, Republic of Korea

**Keywords:** exercise, hypertension, long-distance runner, cardiovascular disease, cardiac death

## Abstract

Sudden cardiac death (SCD) is rare among athletes. However, hypertrophic cardiomyopathy is the leading cause of SCD among those <35 years of age. Meanwhile, coronary artery disease (CAD) is the primary SCD cause among those ≥35 years of age. CAD-induced plaque ruptures are believed to be a significant cause of cardiovascular diseases in middle-aged individuals who participate in extreme long-distance running activities such as marathons. A total of 1970 articles related to EIH were identified using search terms. Out of these, 1946 studies were excluded for reasons such as arterial hypertension, exercise-induced pulmonary hypertension, the absence of exercise stress testing (EST), and a lack of relevance to EIH. The study analyzed 24 studies related to both long-distance runners with exercise-induced hypertension (EIH) and the general public. Among these, 11 studies were quasi-experimentally designed studies used in randomized controlled trials (RCTs) on long-distance runners with EIH. Additionally, 12 studies utilized cohort designs, and one study with a quasi-experimental design was conducted among the general population. Recent studies suggest that an imbalance between oxygen demand and supply due to ventricular hypertrophy may be the actual cause of cardiovascular disease, regardless of CAD. Exercising excessively over an extended period can reduce endothelial function and increase arterial stiffness, which in turn increases afterload and leads to an excessive increase in blood pressure during exercise. Exercise-induced hypertension (EIH), which increases the morbidity rate of resting hypertension and is a risk factor for cardio-cerebro-vascular diseases, is more prevalent in middle-aged long-distance runners than in runners from other age groups, and it increases the prevalence of critical arrhythmias, such as atrial fibrillation or ventricular arrhythmias. EIH is associated with angiotensin II activity, and angiotensin II receptor blockers show promising effects in middle-aged runners. Further, guidelines for preventing excessive participation in races and restricting exercise intensity and frequency would be useful. This review identifies EIH as a potential risk factor for cardiovascular diseases and describes how EIH induces SCD.

## 1. Key Points

Sudden cardiac death (SCD), a result of excessive running in the middle-aged, may be caused by exercise-induced hypertension (EIH).

EIH increases myocardial oxygen demand during exercise, increasing inflammatory reactions and the levels of vasoconstrictors and cardiac markers. In addition, EIH can result in the formation of plaque in the coronary artery and cause fatal arrhythmia, along with atrial expansion and myocardial hypertrophy. 

EIH can cause an imbalance in oxygen supply in the myocardium, rather than coronary artery disease, and act as a new potential risk factor for SCD. 

## 2. Introduction

Cardiovascular disease (CVD) is the leading cause of death worldwide, accounting for approximately 17 million deaths annually. Sudden cardiac death (SCD), defined as unexpected, sudden death, accounts for approximately 25% of cardiovascular mortalities [1]. Regular exercise can reduce the risk of CVD [2], hypertension [3], cardiovascular mortality [4], and glycated hemoglobin levels in diabetic patients [5]. It also improves the physical functioning of patients with heart failure [6], reduces all-cause mortality, and is a benefit to cardiovascular health [7]. Several studies have reported a reduced risk of chronic cardiac disease in long-distance runners, including full- and ultramarathon runners [8], suggesting that long-distance running may effectively reduce the risk of CVD [9]. However, several reports have suggested that excessive exercise may increase the risk of cardiovascular events, such as coronary artery plaque ruptures, and is associated with myocardial infarction and SCD [10,11,12,13]. 

The incidence of SCD among athletes is 0.13–0.75 cases per 100,000 [14]; among middle-aged individuals, the incidence is 6 per 100,000 [15]. Of 215,413 marathon runners in the United States with a mean age of 37 years, studied from 1976 to 1994, four experienced SCD. The incidence of SCD among marathon runners is 1 per 50,000 [11]. These numbers are surprising, as most runners experiencing SCD were found to be experienced athletes. 

Hypertrophic cardiomyopathy (hCMP) and congenital heart defects are the major causes of exercise-induced death (EIN) among those aged <35 years [16,17,18], while ischemic heart disease accounts for more than 70% of all EINs among those aged ≥35 years [16,17,18]. The risk of SCD is 5 to 7 times higher during extreme forms of exercise, such as marathons, than during moderate exercise [8], acting to induce SCD with a history of myocardial ischemia or cardiac arrhythmia [19,20]. Thrombi resulting from exercise-induced atherosclerotic plaque ruptures are a suspected cause of SCD among older athletes [21,22]. Kim et al. also reported that the reason SCD occurs during long-distance running is not due to atherosclerotic plaque ruptures, but due to ischemia resulting from an imbalance in oxygen demand and supply [23]. An increase in oxygen demand is a known cause of myocardial ischemia in patients with CVDs [24]. Exercise-induced hypertension (EIH), or the excessive elevation of blood pressure during exercise, can be defined as a resting SBP and diastolic blood pressure (DBP) < 140/90 mmHg and a maximal SBP ≥210 mmHg in men and ≥190 mmHg in women [25,26].

Recently, long-distance runners with EIH have been found to experience increased myocardial oxygen demand due to excessive increases in blood pressure during exercise [27], leading to an acute increase in the cardiac markers [28] expressed in myocardial infarction. Chronic endothelial dysfunction [29], decreased myocardial diastolic function and myocardial hypertrophy [30], fatal arrhythmia [31], myocardial ischemia on electrocardiograms [32], and, crucially, an increased prevalence of coronary artery plaques [33] have been reported. The results of these studies suggest that EIH in long-distance runners may produce a significant possibility of developing SCD during exercise or competition. There is still no direct evidence that EIH causes SCD in long-distance runners. However, the following research results are already well known in the general population: EIH increases the incidence of resting hypertension [34] and is an independent risk factor for cardio-cerebro-vascular disease (CCVD) [35,36]. Therefore, based on the studies showing a high mortality rate in the general population with EIH, this study describes the need to review the cardiovascular side effects of long-distance runners with EIH and consider the possible mechanism behind SCD induction during excessive exercise.

The purpose of this review is to suggest a potential mechanism for how EIH can lead to cardiovascular disease and assess the possibility of sudden cardiac death by examining various studies. The review will also examine the mechanisms responsible for excessive blood pressure elevations during exercise. In addition, it aims to propose a mechanism that could lead to SCD by reframing the association. This study also discusses the prevention and management of EIH in order to increase stability in middle-aged runners with EIH.

## 3. Methods

We searched articles related to EIH in PubMed and MEDLINE, Google Scholar, and Web of Science and used the Research Information Sharing Service to search for domestic articles. The search keywords were as follows: exercise, hypertension, runner, athletes, cardiovascular disease, and sudden cardiac death. Similar keywords to those related to EIH, such as exaggerated blood pressure response and exercise hypertension, were also used.

The articles were first reviewed based on the abstract, followed by an analysis of methods, results, and discussion sections to understand their content. We compared the results of our previous studies, examining dozens of cases of EIH in middle-aged individuals in relation to those at risk of EIH from 2012 to 2021. The definition of middle-aged in this review is between 40 and 60 years. Previous studies on middle-aged runners with EIH primarily examined marathon and ultramarathon runners.

**Figure 1:**
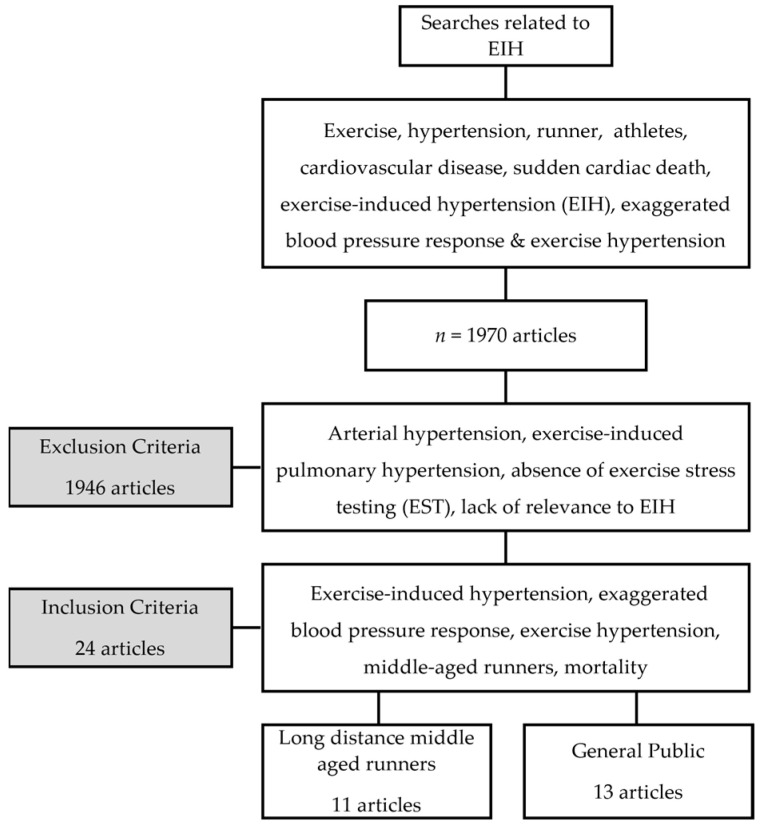
Flow chart of research process.

A total of 1970 articles related to EIH were identified using search terms. Out of these, 1946 studies were excluded for reasons such as arterial hypertension, exercise-induced pulmonary hypertension, the absence of exercise stress testing (EST), and a lack of relevance to EIH. Our research indicated that 11 quasi-experimentally designed studies were conducted in randomized controlled trials (RCTs) on long-distance runners with EIH, as presented in Table 1. Furthermore, Table 2 includes 12 cohort studies and 1 quasi-experimental design for use on the general public. In total, we discussed and analyzed 24 studies related to this topic (Figure 1).

This review investigates the disparities in (dependent variables) among individuals with and without EIH across participant characteristics. It involves longitudinal observations of dependent variables within each group, excluding studies utilizing randomized assignment. The studies involving long-distance runners comprised 11 quasi-experimental designs. A pre-test was initially administered to ensure homogeneity across the two groups. This was followed by a post-test to assess changes between groups. This approach is regarded as a strength of these studies. Out of the 13 studies focused on the general public, one adopted a quasi-experimental design. The cohort study, which spanned the general life course, boasted a larger sample size compared to those targeting long-distance runners, resulting in a lower potential for selection bias. Moreover, its ability to conduct relatively long-term follow-up observations allowed for the examination of enduring effects, a notable strength. However, the study faced the limitation of increased exposure to diverse external factors in real-life settings. Notably, all the studies included in this review shared the advantage of objective assessment, minimizing the potential for subjective interpretation by researchers in blood analysis or the assessment of test results from medical equipment.

### 3.1. Mechanism of EIH in Long-Distance Runners (Figure 2)

A cohort study on various age, sex, and ethnic groups reported an EIH prevalence rate of 3% to 4% [43], while that among healthy middle-aged men was 40% [53]. A recent study of 606 middle-aged long-distance runners reported an EIH prevalence rate of 56% (338), higher than the prevalence found in the general population [35]. A long-distance race such as a marathon requires relatively high cardiorespiratory fitness. To maintain this, one must complete 200–300 metabolic-equivalent task minutes of physical activity, equivalent to 5–10 times the level of physical activity recommended for preventing CVD [54,55]. Repeated training at this exercise level can lead to excessive cardiovascular stress, resulting in myocardial hypertrophy [9,56,57]. An excessive volume and pressure overloads during exercise can also negatively affect arteries [58]. In a normal SBP response to exercise, shear stress on the vascular wall increases as cardiac output increases, and endothelial cells release nitric oxide (NO), triggering vascular dilation in order to maintain stable blood pressure. However, excessive exercise can cause chronic mechanical stress in the arterial walls, impairing endothelial function and increasing peripheral vascular resistance. This increases the afterload and results in excessively elevated blood pressure [59,60]. Endothelial dysfunction, resulting from excessive exercise, induces secondary elastin degeneration due to chronic arterial stress and leads to fibrosis and increased arterial stiffness [37,57]. Tzemos et al. [61] reported the occurrence of endothelial dysfunction due to impaired vasodilation during an exercise stress test in a group that showed excessive elevation of SBP. Stewart et al. [60] reported that impaired vasodilation can excessively increase blood pressure during exercise. Thanassoulis et al. [62] reported that EIH is associated with increased aortic stiffness, and Burr et al. [63] found an association between increased resting SBP and decreased aortic compliance in ultramarathon runners. We recently reported the reduced presence of NO, a vasodilator, in long-distance runners and identified a possible association with angiotensin II activity in the renin–angiotensin–aldosterone system (RAAS) [32]. In a brachial arterial dilation test of middle-aged runners with EIH, the runners exhibited greater arterial stiffness than those with normal blood pressure after exercise, suggesting a potential increase in the risk of CCVD for runners with EIH [38]. The excessive elevation of exercise blood pressure accelerates atherosclerosis in middle-aged men, exacerbating the morbidity and mortality of CVD [30,32,64]. Endothelial dysfunction is a risk factor, requiring attention in long-distance runners with EIH.

**Figure 2:**
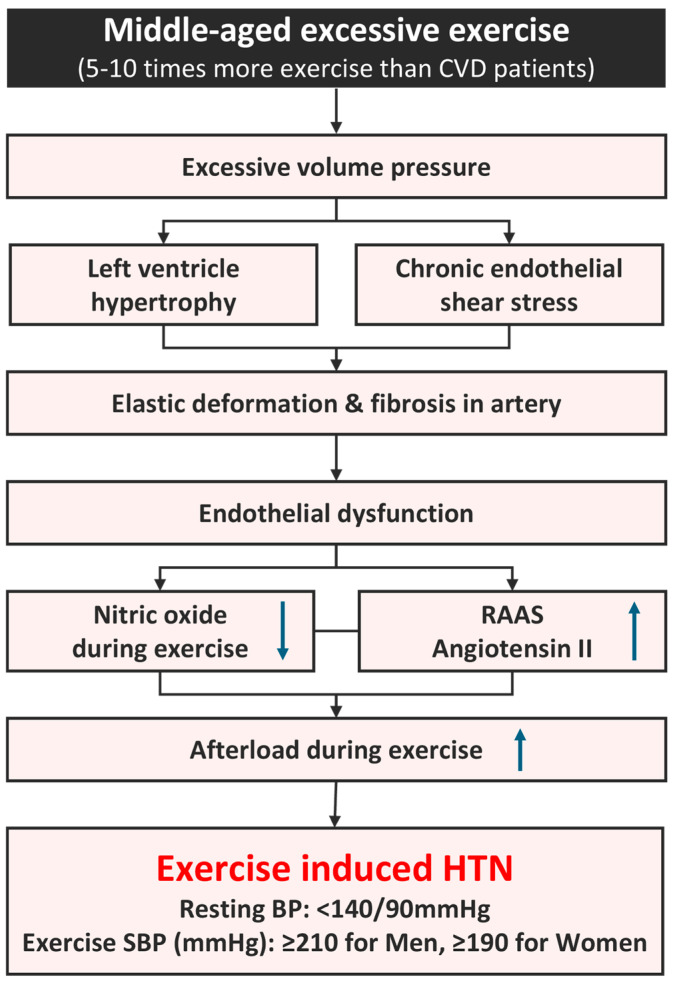
Mechanism of EIH in long-distance runners. BP, blood pressure; CVD, cardiovascular disease; HTN, hypertension; RAAS, renin–angiotensin–aldosterone system; SBP, systolic blood pressure. ↑; increase, ↓; decrease, Refs. [9,25,26,27,37,54,55,56,57,58,59,60].

### 3.2. Atherosclerotic Plaque Development in EIH

Resting hypertension is a risk factor for coronary artery disease (CAD), heart failure, and stroke and increases the risk of SCD [65]. Like resting hypertension, EIH is a risk factor for CAD and cerebrovascular disease [28,29]. A chronic and excessive increase in exercise blood pressure is accompanied by endothelial dysfunction and increased arterial stiffness [58] and triggers inflammatory responses from the endothelial cells that create a favorable environment for plaque formation [31]. Multiple lines of evidence suggest that the excessive elevation of blood pressure causes death from cardiovascular conditions, such as CAD and cerebrovascular disease. This is not only the case in long-distance runners, but also other population groups, including middle-aged males [25,28,29,44,45,46,47,48,49,50,51,52,64,66].

### 3.3. CAD in Long-Distance Runners

Tsiflikas et al. [67] performed cardiac computed tomography (cCT) on 50 middle-aged male amateur marathon runners and detected atherosclerosis in 24. Of these athletes, 20, 3, and 1 showed mild, moderate, and significant CAD, respectively. An important finding of their study was that treadmill tests did not detect multiple cases of CAD, indicating the importance of cCT for CAD detection. Merghani et al. [68] compared the prevalence of CAD between middle-aged master-endurance athletes and normal individuals. They reported a higher prevalence of atherosclerosis among the athletes compared with the control group (44.3% vs. 22.2%). In addition, calcified plaques were common in the athlete group (72.7%), whereas mixed-type plaques were more common in the control group (61.5%). These results indicate that, despite the higher prevalence of atherosclerosis, athletes are less vulnerable to acute myocardial infarction than the general population because they develop stable, calcified plaques. However, this does not mean that such athletes are free from the risk of CAD.

### 3.4. RPP and Oxidative Stress at High Exercise Intensity

A high degree of exercise intensity is an important cause of CAD in long-distance runners. This situation increases the production of reactive oxygen species (ROS), reactive nitrogen species, and oxidative stress [69]. Aging [70], extreme exercise such as ultramarathons [71], and hypertension [72] also increase ROS, oxidative stress, and inflammation, inducing endothelial dysfunction associated with increased arterial stiffness. Vigorous exercise increases the production of free radicals, oxidative stress, and markers of low-density lipoprotein (LDL) oxidation, promoting atherosclerosis [73,74]. In other words, vigorous exercise accelerates atherosclerosis by increasing ROS and oxidative stress, likely causing a significant increase in RPP. These chronic stimuli increase excessive blood pressure during exercise by increasing arterial stiffness and peripheral arterial resistance. In addition, RPP increases as exercise intensity increases due to high blood pressure, even at an appropriate heart rate. Carter et al. [75] stated that the higher the exercise intensity was, the higher the RPP was, and the higher the level of TNF-a (tumor necrosis factor-α) became, an indicator of oxidative stress. These conditions can lead to myocardial hypertrophy due to increased follow-up load, which can eventually trigger CAD in middle-aged runners with EIH.

### 3.5. CAD, Angiotensin II, and Myocardial Ischemia in EIH Runners

We were the first researchers to investigate the prevalence of CAD in both middle-aged runners with EIH and those with normal blood pressure [31]. Consistent with our prediction, middle-aged runners with EIH showed higher coronary artery calcium scores than runners with normal blood pressure. As expected, the EIH group included 12 cases of coronary artery stenosis, while the control group contained only 1. A few patients from the EIH group had coronary artery stenosis greater than 70%, even though the exercise stress test failed to diagnose CAD in any of these cases. One of these patients had undergone percutaneous coronary intervention and another pharmacotherapy. To investigate the mechanism by which EIH occurs in such runners, we examined RAAS and found that angiotensin II is the first hormone to become activated in runners with EIH. Consequently, we confirmed that the existing theory, stating that angiotensin II activity is associated with reduced NO activity, is true in middle-aged runners with EIH [32].

Recently, we detected meaningful ST depression in an exercise stress test involving 606 middle-aged individuals [35]. Overall, 9 subjects in the EIH group and 1 in the normal-exercise group demonstrated ST depression. Based on these results, we determined that excessive exercise, in addition to aging, increases arterial stiffness, which can lead to coronary atherosclerotic plaque formation and is particularly dangerous for those with EIH. In brief, middle-aged runners should undergo both an exercise stress test and also cCT to ensure that they are CAD-free and can run safely.

### 3.6. Cardiac/Inflammatory Markers and Long-Distance Runners with EIH (Table 1)

Major cardiac markers include cardiac troponin I (cTnl) and cTnT, which are expressed in the presence of myocardial infarction caused by myocardial damage [76], and N-terminal pro-B-type natriuretic peptide (NT-proBNP), which is a marker of heart failure [77]. Multiple studies have reported a clinically significant increase in the expression of cardiac markers during and after long hours of extreme exercise [13,78,79,80]. This raises a concern regarding the dangers of extreme exercise and has prompted research into the association between long-distance running and SCD [81].

The level of cardiac troponin has been used as a diagnostic marker of myocardial infarction. It has been theorized that the cardiac troponin level increases during intense exercise due to increased afterload and that this subsequently increases the production of oxygen free radicals that disrupt the cell membrane to induce its release [82].

Another hypothesis suggests that transient ischemia alone can trigger the release of cytoplasm and vesicular materials from the plasma membranes of cardiac muscle cells, even in the absence of myocardial necrosis [83]. This theory has attracted support since Kim et al. [23] reported autopsy results that showed the significant number of SCDs involving ischemic phenomena in long-distance runners, which resulted from an imbalance between oxygen demand and supply rather than atherosclerotic plaque ruptures.

We examined the cardiac and vascular markers, blood parameters, and electrocardiograms (ECGs) of long-distance runners following a resting ECG or exercise stress test in various situations, including marathons; 100 km ultramarathons; and 308 km ultramarathons (Table 1). We observed greater left ventricular mass and a significant reduction in ventricular diastolic function in long-distance runners with EIH compared with hypertensive patients [33]. Although the ventricular diastolic function was not pathologically impaired, a follow-up study examining the effects of a relative reduction in compliance due to left ventricular hypertrophy is necessary.

We also found that marathon runners with EIH experienced a greater increase in levels of endothelin-1, a potent vasodilator, and cTnl compared with runners with normal-exercise blood pressure after completing a race [39]. The excessive elevation of blood pressure observed in the EIH group during a marathon may be attributable to an intercellular calcium overload accompanied by calpain activation and increased oxidative stress, accelerating the exudation and synthesis of the myocardial cell membrane [84]. In addition, we found no differences in the levels of cTnl between the EIH group and the normal-exercise blood-pressure group in the 100 km ultramarathon, unlike the increase in the level of NT-proBNP in the 50 km and 100 km races, which reflects the ventricular myocardial workload at the 50 km and 100 km marks [34]. Additionally, levels of creatinine kinase (CK), which reflects damage to active muscle, and high-sensitivity C-reactive protein (hsCRP), a marker of inflammation, were higher in the 100 km ultramarathon group [34]. These results demonstrate that, while a 100 km ultramarathon race is relatively low-intensity exercise compared with a marathon, the EIH group had a high RPP due to an increase in the NT-proBNP level accompanied by endothelial dysfunction of the musculoskeletal system, resulting in transient ischemia and inflammatory responses, as reflected by the increase in CK and hsCRP levels. In our comparison of soluble vascular cell adhesion molecule-1 (sVCAM-1) level, a value which reflects endothelial damage, in runners who completed a 308 km ultramarathon, we found that the EIH group had an increased sVCAM-1 level at the 100 km and 200 km marks and an increased soluble E-selectin level at the 100 km mark compared with the control group [40]. Despite its low-intensity nature, long-distance running was associated with significant stimulation and a loss of protective effects on endothelial cells in the EIH group. Additionally, we observed a low interleukin-10 level in the resting state in the EIH group following a 100 km ultramarathon and reduced NO production immediately after the completion of the race, suggesting high-blood-pressure elevation due to endothelial dysfunction leading to an increased afterload during exercise [41]. Similarly, high levels of NT-proBNP were observed for up to 24 h after a 100 km ultramarathon [42]. The factors associated with elevated blood pressure were correlated with increases in cTnl, NT-proBNP, endothelin-1, and hsCRP in middle-aged marathon runners in a way that was independent of running history, number of finishes, and peak oxygen uptake [85].

Together, these results, obtained under various conditions, showed increased levels of cardiac markers, inflammatory reactions, and endothelial dysfunction in middle-aged long-distance runners with EIH compared with controls. Moreover, increased blood pressure translates into not only an increase in the RPP, but also into excessive and constant endothelial stimulation that can accelerate atherosclerosis. These results show that runners with EIH are exposed to environments that cause higher risk of SCD development compared with those athletes who train regularly or have normal blood pressure during a run.

### 3.7. Arrhythmogenic Mechanism in Long-Distance Runners with EIH (Figure 3)

#### 3.7.1. Exercise Type and Myocardial Remodeling

Unlike the general population, athletes who participate in extreme training exhibit clear alterations in cardiac structure. Eccentric hypertrophy, which is characterized by an increase in the left ventricular internal dimension without a commensurate increase in myocardial thickness, is characteristically observed in aerobic athletes such as long-distance runners [86]. Eccentric hypertrophies are formed by continuous blood filling and exudation to the heart following long-distance running, with a maximum heart ejection of 40 L and a moderate average blood pressure of 175/69 mmHg [87]. However, anaerobic athletes, such as weightlifters, bodybuilders, and wrestlers, who need to exert a large amount of force in a short period of time, exhibit concentric hypertrophy, characterized by an increase in myocardial thickness rather than in left ventricular internal dimensions [88]. This is due to excessive muscle contraction, which causes momentary high blood pressure and a high stroke volume, producing a large load on the ventricular wall [89]. Rowers or cyclists who utilize both aerobic and anaerobic systems exhibit increases in both myocardial thickness and ventricular dimensions and have the highest left ventricular mass in comparison with other athletes [88]. As a consequence, the causes of the different types of myocardial structure observed can be attributed to the persistent maintenance of high heart rates due to continuous muscular contractions, blood pressure levels approximating 200 mmHg, and a sustained elevation in stroke volume over an extended duration [90]. Heart rate and blood pressure affect myocardial remodeling according to the type of exercise, and excessively high blood pressure during exercise is the main factor causing this deformation [90,91,92].

**Figure 3:**
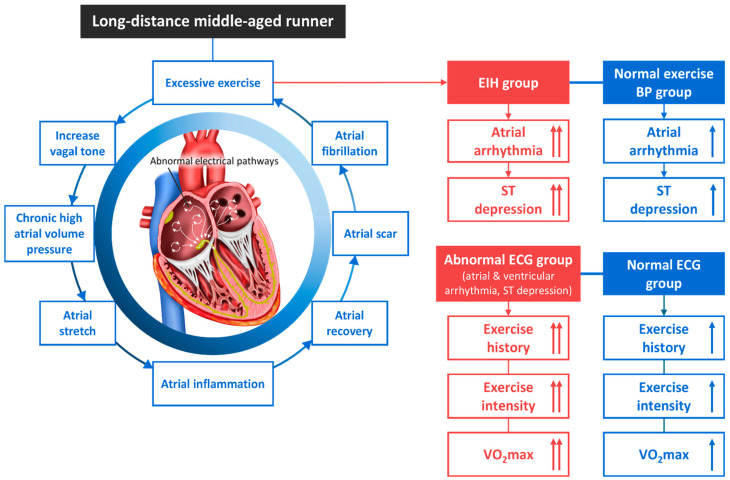
The atrial fibrillation mechanism and an abnormal electrocardiogram of EIH in middle-aged long-distance runners. BP, blood pressure; ECG, electrocardiogram; EIH, exercise-induced hypertension. ↑; increase, Refs. [31,32,56,91,93,94,95,96,97].

#### 3.7.2. Chronic Excessive Exercise and AF

Athletes often experience volume and pressure overloads due to the frequent need to exert a maximal force, which pushes a large amount of blood back into the atria and causes the heart to contract. As the atria and ventricles experience a chronic workload, they thicken in order to adapt. Further, when the atria are exposed to high volumes and pressure loads for an extended period due to excessive exercise, an increased vagal tone can develop and the atria can stretch. With repeated inflammation and repair, an atrial scar can form and progress to fibrosis, resulting in atrial arrhythmias such as atrial fibrillation (AF) [91]. While walking or moderate-intensity running reduces the prevalence of cardiac arrhythmia in the general population [93], excessive endurance exercise can induce critical arrhythmias such as atrial flutter/AF and ventricular tachycardia [56,94,95,98]. The risk of AF is reportedly 5 times higher among endurance athletes than the general population [96,97]. Mont et al. [99] reported that carrying out at least 3 h of exercise per week for more than 10 years is associated with AF. Karjalainen et al. [97] reported that the incidence of AF increases after 1500 h of exercise. The occurrence of AF is a risk factor that increases SCD in people with coronary artery disease, myocardial infarction, heart failure, Brugada syndrome, and hypertrophic cardiomyopathy, as well as in the general population [100,101]. Therefore, early detection is important because AF, which is caused by chronic excessive exercise or prolonged exposure to EIH, can cause SCD.

#### 3.7.3. AF in Middle-Aged Runners with EIH

Our previous study of 552 middle-aged runners used the results of exercise stress tests to divide the cohort into a critical arrhythmia group and a normal group. Both groups had EIH, and the arrhythmia group had higher exercise intensity and VO_2max_, as well as a more intense training history, compared with the normal group [36].

In our recent study, we divided 606 middle-aged runners into an EIH group and a non-EIH group and compared them. We found that runners with an abnormal ECG response (AER) had a training history and exercise time much longer than those with a normal ECG response (non-AER). The incidence of atrial arrhythmia and significant ST segment depression was higher in the EIH group than in the non-EIH group. The incidence of AER was significantly higher in the EIH group than in the non-EIH group [35].

In both studies, we observed 14 middle-aged runners (2.5%) with critical arrhythmias (10 with AF and 4 with non-sustained ventricular tachycardia) [36]. In addition, we found that middle-aged long-distance runners with AER had a longer training history and total exercise time than those without, and that the EIH group had a higher incidence of AER than the non-EIH group [35]. These results indicate that EIH is a risk factor for critical cardiac arrhythmias in middle-aged runners participating in extreme exercise. Follow-up research is recommended.

### 3.8. SCD Hypothesis in Middle-Aged Runners with EIH (Figure 4)

Hypertension is an independent risk factor for 50% of all cases of coronary heart disease and two-thirds of all stroke cases [102]. Hypertension also induces left ventricular hypertrophy (LVH) and is an independent risk factor for ventricular arrhythmias [103] and SCD [65,104]. Recent studies have reported that EIH is a risk factor for SCD in healthy individuals (Table 2) [45,105]. In this review, we propose several hypotheses regarding the mechanism of SCD in middle-aged runners with EIH based on these existing theories.

**Figure 4:**
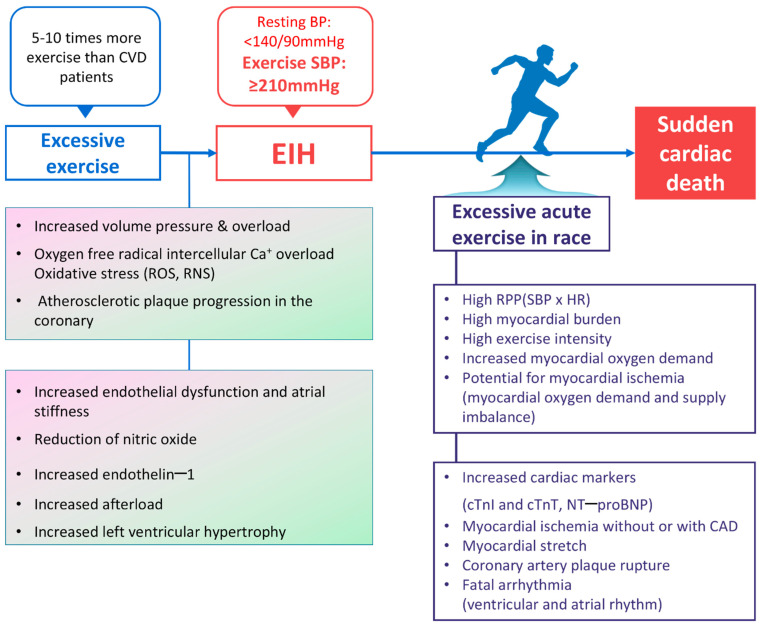
Summary of the sudden cardiac death mechanism hypothesis in middle-aged long-distance runners with EIH. BP, blood pressure; CAD, coronary artery disease; cTnI, cardiac troponin I; cTnT, cardiac troponin T; NT–proBNP, N–terminal pro–B-type natriuretic peptide; CVD, cardiovascular disease; EIH, exercise-induced hypertension; HR, heart rate; RNS, reactive nitrogen species; ROS, reactive oxygen species; RPP, rate pressure product; SBP, systolic blood pressure. Refs. [10,11,12,13,21,22,23,24,27,28,31,32,33,37,38,39,41,43,44,56,58,59,60,61,68,71,75,76,77,78,79,80,82,85,91,93,94,95,96,97,106,107,108,109].

First, moderate levels of aerobic exercise lower inflammatory precursors and improve endothelial function, increasing nitrate levels and controlling resting blood pressure in hypertensive patients [110]. However, excessive exercise increases oxidative stress [71], damaging and impairing endothelial cells and increasing arterial stiffness [58]. This process increases afterload during exercise and, if it becomes chronic, can increase the prevalence of resting hypertension [27]. Progression to EIH leads to potential coronary atherosclerotic plaque formation and is highly likely to result in SCD due to asymptomatic ischemia or acute plaque ruptures [31,35]. When conducting studies on asymptomatic ischemia in athletes, Chevalier et al. [106] found 24 cases (1.7%) of cardiovascular disease among 1361 asymptomatic middle-aged athletes, and Katzel et al. [107] reported discovering asymptomatic ischemia in the exercise tests graded the highest and tomographic thallium scintigraphy tests of master athletes (60 ± 6 years, *n* = 70). More than half of SCD cases during exercise occur in asymptomatic individuals unaware of their condition [111].

Second, while hypertension-induced myocardial hypertrophy is a risk factor for SCD [65], the main cause of SCD during exercise is hCMP [18]. Although we could not confirm that EIH-induced myocardial hypertrophy is a direct cause of SCD [108], middle-aged runners with EIH can lack myocardial perfusion during exercise compared with athletes with normal hearts. In addition, the excessive-related elevation of blood pressure is associated with LVH [44]. High blood pressure can increase RPP and myocardial oxygen demand in runners with LVH during exercise, leading to an imbalance between oxygen demand and supply. In runners with EIH and LVH, increased RPP induces transient ischemia; the ventricles stretch excessively due to overloading in terms of volume and pressure, inducing ectopic activity and, consequently, ventricular tachycardia [112]. In addition, myocardial hypertrophy and volume/pressure overload, capable of inducing myocardial ischemia, are particularly dangerous for runners with potential atherosclerotic plaques. A recent study reported finding a higher prevalence of coronary atherosclerotic plaques in middle-aged runners compared with the general population [68]. This study is able to hypothesize that ischemia, resulting from an imbalance between oxygen demand and supply in the coronary arteries, is a more likely cause of SCD among runners compared with ruptures in the coronary artery [23].

Third, excessive exercise exposes the heart to volume and pressure overload, which increases the prevalence of atrial enlargement and inflammation, healing, fibrosis, and AF [91]. EIH is likely to accelerate these conditions. Athletes who perform excessive amounts of exercise are between 2.5 and 5 times more likely to have AF than the general population inside Korea [35] and outside Korea [96,97], respectively. AF is highly likely to induce cerebral or myocardial infarction due to cardio-cerebro-vascular embolisms because it promotes thrombosis. Patients may become aware of AF through symptoms such as palpitations and an irregular pulse. While AF may be safe if treated early, it can lead to SCD during exercise if left unrecognized.

Although age is a risk factor for CVD, adequate exercise can mitigate the risk of CVD among the elderly. However, excessive and chronic exercise damages endothelial cells and increases arterial stiffness, increasing afterload during exercise and inducing EIH. Many reports suggest that EIH can further impair endothelial function and increase the RPP during exercise due to myocardial hypertrophy. Additionally, it may induce coronary atherosclerotic plaque formation or rupturing. Based on all these mechanisms, it can be hypothesized that EIH may cause lethal ischemia due to an imbalance between myocardial oxygen demand and supply. Furthermore, there is a possibility that EIH may increase the risk of SCD and serve as a significant risk factor for SCD in middle-aged runners.

### 3.9. Can EIH Be Improved by Exercise?

When treating the general public, motor-induced hypertension is likely to be sufficiently improved through regular exercise because the main cause of the illness is an increase in arterial stiffness due to endothelial cell dilatation-related dysfunction of the artery. However, limited regular exercise has been proven effective in treating people with EIH. In previous studies into middle-aged men who exercised regularly, the increase in blood pressure was stable during exercise compared to the non-exercise group, and there was no excessive increase in blood pressure [113]. In cardiovascular patients with EIH, 14-week cardiac rehabilitation significantly reduced maximal systolic blood pressure [114]. Furthermore, this cardiac rehabilitation showed that regular aerobic exercise had a distinct antihypertensive effect in African Americans with excessive blood pressure during exercise [109,115]. This phenomenon shows that regular aerobic exercise has an antihypertensive effect on subjects with excessive blood pressure increases during exercise, as well as on blood pressure when stabilized. However, most of the subjects in that study had no exercise experience. As shown in Table 2, subjects with excessive blood pressure increases have a higher mortality rate than those with normal blood pressure during exercise. It is possible that regular exercise can reduce future mortality in subjects with EIH, but research on this topic is insufficient. In this study, It is difficult to expect anticipate the coercive effect of EIH through exercise in veteran long-distance runners who already have EIH. Indeed, the stronger such athletes exercise, the more likely it is to worsen. The effect of exercise intensity control or drug control should be verified in endurance athletes.

### 3.10. Interventions for Prevention of SCD in Runners with EIH

Middle-aged long-distance runners with EIH engage in up to 10 times the recommended level of physical activity for patients with CVD. As we mentioned before, EIH that results from excessive exercise in middle-aged individuals who participate in high-intensity exercise induces oxidative stress and endothelial dysfunction, increases arterial stiffness, and induces ventricular hypertrophy. It also increases cardiac marker levels and induces early hypertension, atherosclerosis, and arrhythmias, leading to SCD. Several interventions are necessary to prevent and treat EIH.

First, oxidative stress and LDL oxidation induce endothelial dysfunction [116], and excessive endurance training, which is a major cause of atherosclerotic lesions [117], can increase LDL oxidation markers [73]. Extensive research has been conducted on the effects of antioxidant-based interventions on the prevention of oxidative stress caused by excessive exercise [118]. There are conflicting results regarding the effects of such interventions, with some researchers reporting the positive effects of antioxidants (vitamin C and E and coenzyme Q10) in individuals who exercise excessively [15,119,120,121,122], and others reporting a lack of changes [123,124,125,126,127,128]. The existing evidence is insufficient to draw conclusions regarding the effectiveness of antioxidant-based interventions [69]. In the absence of a well-established guideline specifying appropriate antioxidant doses for the general population and for long-distance runners, it is difficult to set criteria regarding antioxidant doses for runners with EIH. We can only predict that long-distance runners with EIH will experience increased oxidative stress as they exercise more frequently than runners with normal blood pressure due to increased RPPs. Further research is needed on levels of oxidative stress and the effects of antioxidants on runners with EIH.

Second, extensive research has been conducted on drugs that reduce EIH, including beta blockers (BBs), which effectively lower SBP during exercise [129,130,131]. BBs are more effective than angiotensin-converting enzyme inhibitors (ACEi), calcium-channel blockers (CCBs), and diuretics in lowering blood pressure during exercise [132]. However, BBs can cause side effects in well-trained amateur marathon runners, who likely have lower heart rates than the general population. Among the various ARBs examined, Olmesartan released the most NO by up to 30% [133]. It has been reported that ARB treatment could lower the incidence of atherosclerosis and cardiovascular disease by lowering arterial stiffness and improving endothelin dysfunction [134]. As a result, Kim et al. [135] recommended angiotensin II receptor blockers (ARBs) and ACEi for patients with EIH because the activation of angiotensin II in the RAAS has been observed in patients with EIH [136]. Warner et al. [137] also reported that ARBs reduce the maximal SBP by 33 mmHg in patients with an excessive blood pressure response. In our recent study, we observed high angiotensin II activity in the RAAS following an exercise stress test in middle-aged runners with EIH [32]. Our study revealed that ARBs can effectively reduce the SPB seen following maximal exercise. Further research is needed to verify the clinical effects of ARBs in middle-aged runners with EIH.

Third, to achieve healthy living, it is recommended that adults exercise for 20–60 min per day, 3–5 times a week, at a VO_2peak_ between 40% and 80% [138]. Long-distance runners have physical activity levels 5–10 times those of patients with CVD [54,55]. They must adjust their exercise intensity according to whether they are active or off-season. Middle-aged runners participating in long-distance races such as marathons or ultramarathons should limit the number of races they run in 1 year and adjust their regular training accordingly. As there are no guidelines for work rate, research that can help identify safe exercise habits is necessary.

Fourth, middle-aged long-distance runners must check their exercise blood pressure at least once every year via an exercise stress test. The recent meta-analysis by Cuspidi et al. [139] reported that individuals with EIH face an increased risk of masked hypertension. This finding suggests that an exaggerated blood pressure response to exercise could serve as a forewarning of cardiovascular risk associated with blood pressure, regardless of any prior diagnosis of heart disease [140]. Therefore, if patients have EIH, they must undergo cardiac CT to verify the existence of CAD. Cardiac CT is strongly recommended for middle-aged long-distance runners, as an exercise stress test alone may be insufficient to the task of detecting CAD.

### 3.11. Research Limitations and Future Research Directions

Although numerous cross-sectional and longitudinal studies have been performed to examine future CAD or mortality in the general population or patients with EIH, the evidence for clear SCD in long-distance runners with EIH is lacking. However, long-distance runners with EIH show greater association with CAD than normotensive runners, and this is the first study based on an important explanation for SCD during exercise or competition. To conclusively establish this, we need to investigate the pathogenesis of EIH runners through large-scale follow-up studies and verify the association between the prevalence of CAD and SCD.

## 4. Conclusions

The incidence of EIH can progress to an imbalance between blood supply and demand due to ventricular hypertrophy, critical arrhythmias such as AF due to atrial enlargement, and coronary atherosclerotic plaque formation, all of which can result in SCA during exercise. However, since there is no conclusive evidence that all of these issues cause SCD during exercise, we propose a potential mechanism behind its development and emphasize the necessity of conducting further research in the future.

## Figures and Tables

**Table 1:** Cardiac markers, blood parameters, cardiovascular side Effects, and Training Characteristics of Middle-Aged Long-Distance Runners with Excessive Blood Pressure Elevation.

Ref	Aim	Group (*n*, age)	Method	Result	Conclusion
Kim et al. [31]	To investigate the prevalence of CAD in middle-aged runners with EIH.	NBPG (*n* = 22, 51.7 ± 4.9) EIHG (*n* = 28, 54.5 ± 5.0)	Comparison of CAD prevalence in EIH and non-EIH groups according to GXT results	EIHG had higher CAC scores (42.6 ± 67.8) than NBPG (2.8 ± 6.0). EIHG had a higher CAC score distribution than NBPG. EIHG had a higher prevalence of coronary artery plaque and maximum internal arterial stenosis than NBPG.	Middle-aged runners with EIH are associated with an increased prevalence of coronary artery plaques. EIH screening via GXT is recommended, followed by MDCT.
Kim et al. [32]	To assess the association between middle-aged runners with EIH and ATII.	NBPG (*n* = 21, 53.6 ± 4.9) EIHG (*n* = 35, 53.9 ± 4.8) CHG (*n* = 14, 52.1 ± 5.1)	Blood collection before and after GXT, followed by RAAS and NO tests	ATII activity and a reduction in NO were associated with endothelial dysfunction in runners with EIH.	ATII inhibitors are appropriate antihypertensive medications for runners with EIH.
Kim et al. [34]	To compare cardiac markers and inflammation before and after a 100 km run between EIH and NOR runners.	NOR (*n* = 10, 46.8 ± 1.2) EIH (*n* = 10, 47.5 ± 1.3)	Blood collection pre-run and at 50 and 100 km	CK: Increase in EIH with respect to NOR at 100 km NT-proBNP: Increase in EIH with respect to NOR at 50 and 100 km CRP: Increase in EIH with respect to NOR at 100 km	Runners with EIH did not show myocardial damage following the 100 km run but had myocardial stress and active muscle damage due to epithelial dysfunction.
Kim et al. [35]	To investigate the prevalence of abnormal ECG response and training characteristics of middle-aged runners with EIH.	NEIHg (*n* = 268, 48.4 ± 7.1) EIHg (*n* = 338, 49.9 ± 7.2) Non-AERg (*n* = 569) AERg (*n* = 37)	Comparison of abnormal responses in GXT and training characteristics	EIHG had higher frequencies of ST segment depression and atrial arrhythmias than NEIHg. AERg had longer training history and greater total training time than non-AERg	The high incidence of myocardial ischemia and atrial arrythmias among middle-aged runners with EIH is associated with excessive training.
Kim et al. [36]	To compare hemodynamic response and training characteristics between the abnormal response group (ARG) and the normal response group (NRG).	NRG (*n* = 538, 49.0 ± 7.3) ARG (*n* = 14, 49.2 ± 7.9)	Comparison of arrhythmias detected in GXT, hemodynamic response, and training characteristics	Both groups had prehypertension and EIH in the resting state. ARG had lower DBP and higher VO_2max_, a longer training history, and higher training intensity than NRG.	Middle-aged long-distance runners exhibited prehypertension and EIH. ARG has high training intensity, a long training history, and high cardiorespiratory fitness and requires regular cardiovascular tests and adequate exercise prescriptions.
Kim et al. [37]	To investigate excessive exercise habits, resting and exercise blood pressure, and cardiac events.	NBPG (*n* = 214, 49.1 ± 7.7) HBPG (*n* = 357, 48.8 ± 6.6)	Comparison of training characteristics and cardiac events between a resting hypertension group and a non-resting-hypertension group based on GXT results	HBPG had a shorter marathon history than NBPG but had higher training intensity, a longer daily training duration, and a higher drinking frequency.	High training intensity and long training time can be used as new indices for potential resting and exercise hypertension for middle-aged long-distance runners.
Yoon et al [38]	To investigate the arterial stiffness of middle-aged runners with EIH	NBPG (*n* = 17, 49.9 ± 5.3) EIHG (*n* = 39, 51.7 ± 5.0) CHG (*n* = 10, 50.8 ± 3.3)	Comparison of AIX, AIX@75bpm, and PWV between groups assigned based on GXT results	CHG had higher AIX and AIX@75bp than EIHG and NBPG. EIHG had higher AIX and AIX@75bp than NBPG. VO_2max_ was inversely correlated with MSBP during exercise, PWV, AIX, and AIX75@bpm	High arterial stiffness can increase the risk of cerebrovascular diseases in runners with EIH. On the other hand, cardiopulmonary fitness is negatively correlated with exercise blood pressure and arterial stiffness. Further research on this correlation is necessary.
Kim et al. [39]	To compare cardiac markers and ET-1 before and after a marathon between EIH and CON runners.	CON (*n* = 10, 52.5 ± 7.9) EIH (*n* = 10, 50.6 ± 7.5)	Blood collection pre- and post-run	cTnI, NT-proBNP, ET-1: Increased in EIH with respect to CON immediately after the run	Increased vascular tone and blood pressure during a marathon increased myocardial stress and perfusion in runners with EIH.
Jee et al. [40]	To compare inflammatory precursors in endothelial cells per section of a 308 km run between EIH and CON runners.	CON (*n* = 8, 49.7 ± 5.6) EIH (*n* = 8, 46.7 ± 5.4)	Blood collection pre-run and at 100 and 200 km	sVCAM-1: Increased in EIH with respect to CON at 100 and 200 km sE-selectin: Increased in EIH with respect to CON at 100 km Leukocytes: Increased in EIH with respect to CON at 308 km	Vascular resistance and shear force can increase during a 308 km run, damaging endothelial cells in runners with EIH.
Kim et al. [41]	To compare anti-inflammatory precursors and NO before and after a 100 km run between EIH and NCG runners.	NCG (*n* = 8, 53.5 ± 8.8) EIHG (*n* = 10, 53.7 ± 4.3)	Blood collection pre-run and at 100 km	IL-10: Decreased in EIH with respect to NCG in the resting state NO: Decreased in EIH with respect to NCG at 100 km	In EIHG, excessive exercise inhibits NO release by endothelial cells, causing blood pressure to rise excessively due to vascular constriction.
Park et al. [42]	To compare cardiac and inflammatory markers in the recovery phase following a 100 km run between EIH and NEBPR runners.	NEBPR (*n* = 11, 51.7 ± 4.3) EIH (*n* = 11, 52.9 ± 3.8)	Pre, 100 km	CK, nTnI: Increased in EIH with respect to NEBPR at 100 km and 24 h NT-proBNP: Increased in EIH with respect to NEBPR at 100 km and 24 and 72 h hsCRP: Increased in EIH with respect to NEBPR at 24 h	Increased inflammation and cardiac markers until the recovery phase can lead to volume and pressure overloads and restricted blood flow in the heart, leading to myocardial damage in runners with EIH.

EIH, exercise-induced hypertension; NOR, normal; CK, creatinin kinase; NT-proBNP, n-terminal pro-brain natriuretic peptide; ET-1, endothelin-1; CON, control; sVCAM-1, soluble vascular cell adhesion molecule-1; sE-selectin, soluble E-selectin; NEBPR, normal-exercise blood pressure response; hsCRP, high-sensitivity C-reactive protein; NCG, normal control group; IL-10, interleukin-10; NO, nitric oxide; GXT, graded exercise test; NRG, normal runners group; ARG, arrhythmic runners’ group; NBPG, normal blood pressure group; HBPG, high-blood-pressure group; CHG, complex hypertension group; EIHG, exercise induced-hypertension group; RAAS, renin–angiotensin–aldosterone-system; MDCT, multi-detector computed tomography; CAC, coronary artery calcium; AERg, abnormal ECG response group; AIX, augmentation index; PWV, pulse wave velocity.

**Table 2:** Studies on Excessive Blood Pressure Elevation During Exercise and Its Associated Cardiovascular Risks and Deaths.

Ref.	Aim	Subject	Method	Result	Conclusion
Allison et al. [25]	To assess the prognosis of subjects with exercise hypertension.	A total of 150 healthy subjects	7.7 ± 2.9-year follow-up	High risk of major cardiovascular death in the EIH group.	Healthy, asymptomatic subjects might be at a high risk of major cardiovascular events.
Kurl et al. [28]	To examine the association between exercise SBP and the risk of stroke.	A total of 1026 subjects without cerebrovascular disease.	10.4-year follow-up	The risk of stroke and ischemic stroke increased with MSBP in the exercise test and blood pressure at 2 min into the recovery phase increased.	Exercise SBP tests are recommended as additional tools in predicting future strokes.
Mundal et al. [29]	To investigate whether casual blood pressure and exercise SBP can predict the morbidity and mortality rates of myocardial infarction in healthy men.	Healthy males (*n* = 1999, 40–59 years). 31,984 patients	Sixteen-year follow-up for males who were healthy from 1972 to 1975	Blood pressure during exercise is a stronger predictor of morbidity and mortality than casual blood pressure due to myocardial infarction.	Blood pressure values measured during an exercise stress test can be used to differentiate between moderate and severe hypertension.
Le et al. [43]	To investigate the association between exercise blood pressure response and CVD.	3045 men (*n* = 1437, 43 ± 10 years) and women (*n* = 1608, 43 ± 10 years)	Measure blood pressure at stage 2 of the Bruce protocol and blood pressure during the recovery phase 20-year follow-up	Increases in SBP and DBP in the exercise stress test were associated with SVD	Low-intensity exercise and DBP in the recovery phase predicted cardiovascular disease in middle-aged adults. Exercise DBP can be a better predictor of exercise SBP.
Gottdiener et al. [44]	To assess the correlation between excessive blood pressure response during exercise in the absence of hypertension and LVH.	Health examination subjects (*n* = 39, male, 44.6 ± 8.5)	SBP ≥ 210 mmHg, SBP < 210 mmHg during maximal exercise	LVH detected in 14 of 22 men with SBP ≥ 210 mmHg. LVH was mild but accompanied by an increase in left atrium size, suggestive of impaired left ventricular filling.	Excessive blood pressure responses during an exercise stress test are associated with LVH, even in non-hypertensive subjects.
Jae et al. [45]	To investigate the association between MSBP during an exercise stress test and the risk of SCD in men.	Health examination subjects from 1984 to 1989 (*n* = 2410, 42–61 years) divided into those with CVD (*n* = 884) and those without (*n* = 1526) Investigate the correlation between MSBP (≥210 mmHg) and SCD in these subjects	Within 1–24 h after the onset of SCD symptoms, analyze data about ventricular tachycardia, atrial fibrillation, and SCD.	For men without a history of CVD, the risk of SCD is high when MSBP ≥ 210 mmHg. For men with a history of CVD, the risk of SCD is high when MSBP < 210 mmHg.	For men without a history of CVD, SCD contributes to endothelial dysfunction, inflammation, atherosclerosis, and future hypertension For men with a history of CVD, SCD is associated with reduced cardiac function
Kjeldsen et al. [46]	To investigate SBP during a cycle ergometer test and cardiovascular mortalities.	A total of 1999 healthy subjects (40–59 years)	21-year follow-up	An excessive increase in SBP measured during moderate-load exercise predicts CV regardless of age, office blood pressure, and other risk factors for CV	An abnormal increase in exercise SBP can warn clinicians of a possible increase in cardiovascular risk regardless of office blood pressure
Kohl et al. [47]	To examine the correlation between hemodynamic response to maximal exercise, CVD, and CHD mortality.	*n* = 20,387 (male, 42.2 years) *n* = 6234 (female 41.9 years)	Mean follow-up of 8.1 years	In total, 348 men and 66 women died during the follow-up period. All-cause mortalities increased as MSBP increased and maximum heart rate decreased.	Excessive SBP or reduced heart rate during maximal exercise increases mortalities.
Laukkanen et al. [48]	To investigate the association between the risk of AMI and exercise SBP response.	A total of 1731 middle-aged subjects without CAD	12.7-year follow-up	Maximal SBP elevation in a cycle ergo test is associated with the risk of myocardial infarction	The speed and extent of SBP elevation in a graded exercise test are predictors of myocardial infarction. Regular blood pressure measurements through exercise tests are important
Schultz et al. [49]	To review the relationships between EIH and cardiovascular events and mortality prediction.	A total of 46,314 subjects without serious CAD	15.2 ± 4.0 years of follow-up	For every 10 mmHg increase in exercise SBP, the risk of cardiovascular events and mortality increased by 4% regardless of office blood pressure, age, and risk factors of CV.	As a risk factor, EIH is independent of CV events and deaths.
Skretteberg et al. [50]	To investigate whether exercise SBP is associated with CHD.	Men (*n* = 1392, mean 49.2 years)	Monitor changes in SBP during a cycle ergo test to assess the risk of CHD and SCD over 7 years.	An increase in exercise SBP over 7 years independently predicts an increase in mortality and the risk of CV and CDH.	High exercise SBP is a strong risk factor for CV in men. The regulation of excessive exercise blood pressure is necessary.
Weiss et al. [51]	To investigate whether excessive blood pressure elevation during exercise increases the risk of cardiovascular death.	Follow-up of 6578 asymptomatic patients (mean age 46 years) who showed excessive blood pressure elevation in Bruce stage 2 and a normal blood pressure group.	GXT (Bruce protocol) followed by a 20-year follow-up	Maximum exercise blood pressure in Bruce stage 2 is significantly associated with death from CVD.	It is necessary to screen non-hypertensive patients with Bruce stage 2 blood pressure > 180/90 mmHg who are at high risk of death from CVD and properly manage them.
Gupta et al. [52]	To investigate whether an increase in SBP is associated with CV prognosis in EST.	6145 men Comparison between a group with SBP elevation ≤ 43 mmHg (*n* = 3062, 61 ± 11 years, A group) and a group with SBP elevation ≥ 44 mmHg (*n* = 3083, 57 ± 11 years, B group)	Mean follow-up of 6.6 years for two groups	676 patients died from cardiovascular conditions during the follow-up period. Higher cardiovascular mortality was found in Group A than in Group B (13.7% vs. 8.2%, *p* < 0.001).	For EST, a ≥44 mmHg increase in SBP was associated with a 23% increase in survival rate

LVH, left ventricular hypertrophy; SBP, systolic blood pressure; EIH, exercise-induced hypertension; CV, cardiovascular; CVD, cardiovascular disease; CHD, coronary heart disease; MSBP, maximal systolic blood pressure; GXT, graded exercise test; SCD, sudden cardiac death.

## Abbreviations

ACEi	angiotensin-converting enzyme inhibitors
AERg	abnormal ECG response group
AF	atrial fibrillation
AIX	augmentation index
ARBs	angiotensin II receptor blockers
ARG	arrhythmia runners’ group
BBs	beta blockers
CAC	coronary artery calcium
CCBs	calcium-channel blockers
cCT	cardiac computed tomography
CCVD	cardio-cerebro-vascular disease
CK	creatinine kinase
CHD	coronary heart disease
CON	control
cTnI	cardiac troponin I
CVD	cardiovascular disease
ECGs	electrocardiograms
CHG	complex hypertension group
EIH	exercise-induced hypertension
ET-1	endothelin-1
GXT	graded exercise test
HBPG	high-blood-pressure group
hCMP	hypertrophic cardiomyopathy
hsCRP	high-sensitivity C-reactive protein
IL-10	interleukin-10
LDL	low-density lipoprotein
LVH	left ventricular hypertrophy
MDCT	multi-detector computed tomography
MSBP	maximal systolic blood pressure
NCG	normal control group
NBPG	normal blood pressure group
NEBPR	normal-exercise blood pressure response
NO	nitric oxide
NOR	normal
NT-proBNP	N-terminal pro-B-type natriuretic peptide
NRG	normal runners’ group
PWV	pulse wave velocity
RAAS	renin–angiotensin–aldosterone system
ROS	reactive oxygen species
RPP	rate pressure product
SBP	systolic blood pressure
SCD	sudden cardiac death
sE-selectin	soluble E-selectin
sVCAM	soluble vascular cell adhesion molecule
